# Conservation and divergence of expression of *GA2-oxidase* homeologs in apple (*Malus* x *domestica* Borkh.)

**DOI:** 10.3389/fpls.2023.1117069

**Published:** 2023-04-26

**Authors:** Songwen Zhang, Christopher Gottschalk, Steve van Nocker

**Affiliations:** Department of Horticulture and Graduate Program in Plant Breeding, Genetics and Biotechnology, Michigan State University, East Lansing, MI, United States

**Keywords:** gibberellin, GA2-oxidase, apple, floral induction, gene duplication, gene expression

## Abstract

In domesticated apple (*Malus* x *domestica* Borkh.) and other woody perennials, floral initiation can be repressed by gibberellins (GAs). The associated mechanism is a major unanswered question in plant physiology, and understanding organismal aspects of GA signaling in apple has important commercial applications. In plants, the major mechanism for elimination of GAs and resetting of GA signaling is through catabolism by GA2-oxidases (GA2ox). We found that the GA2ox gene family in apple comprises 16 genes representing eight, clearly defined homeologous pairs, which were named as *MdGA2ox1A/1B* to *MdGA2ox8A/8B*. Expression of the genes was analyzed in the various structures of the spur, where flowers are initiated, as well as in various structures of seedlings over one diurnal cycle and in response to water-deficit and salt stress. Among the results, we found that *MdGA2ox2A/2B* dominated expression in the shoot apex and were strongly upregulated in the apex after treatment with exogenous GA_3_, suggesting potential involvement in repression of flowering. Several *MdGA2ox* genes also showed preferential expression in the leaf petiole, fruit pedicel, and the seed coat of developing seeds, potentially representing mechanisms to limit diffusion of GAs across these structures. In all contexts studied, we documented both concerted and distinct expression of individual homeologs. This work introduces an accessible woody plant model for studies of GA signaling, *GA2ox* gene regulation, and conservation/divergence of expression of homeologous genes, and should find application in development of new cultivars of apple and other tree fruits.

## 1 Introduction

Gibberellins (GAs) are a class of phytohormones found in all vascular plants. To date, a total of 136 GA forms have been identified, among which GA_1_, GA_3_, GA_4_ and GA_7_ are the well-studied bioactive forms in plants (reviewed in Hedden, 2020). The early steps of GA biosynthesis involve the production of *ent*-kaurene from trans-geranylgeranyl diphosphate (GGPP) by *ent*-copalyl diphosphate synthase (CPS) and *ent*-kaurene synthase (KS), and the subsequent generation of the initial GA form, GA_12_, by *ent*-kaurene oxidase (KO) and *ent*-kaurenoic acid oxidase (KAO). The later steps involve sequential oxidation of non-bioactive, 20-carbon (C20) GA precursors to generate active C19 GAs, accomplished by two groups of 2-oxoglutarate-dependent dioxygenases (2-ODDs), GA20-oxidase (GA20ox) and GA3-oxidase (GA3ox).

In angiosperms and gymnosperms, both C20 and C19 GAs can be catabolized by a distinct group of 2-ODDs, the GA2-oxidases (GA2ox). In *Arabidopsis thaliana* (Arabidopsis), where the genetics of GA biosynthesis have been most extensively studied, GA2ox is thought to limit the accumulation of bioactive GAs, both through diversion of early intermediates from the pathway and through catabolism of bioactive forms. Although alternative pathways for inactivation of GAs have been described (Schneider and Schliemann, 1994; Zhu et al., 2006; Varbanova et al., 2007), GA2ox catabolism appears to be the predominant route for the elimination of bioactive GAs and resetting of GA signaling. In plant tissues, appropriate levels of GAs are maintained through a homeostatic mechanism of biosynthesis and catabolism, which involves the GA-dependent repression (*GA20ox*, *GA3ox*) and activation (*GA2ox*) of gene expression.

GA2ox enzymes (gibberellin 2-β-dioxygenases; EC 1.14.11.13) comprise three distinct classes (Lee and Zeevaart, 2005; Serrani et al., 2007). Class I and II GA2ox specifically target C19 GAs for hydroxylation at C-2 (Rieu et al., 2008). Class III enzymes target the early intermediate C20-GAs, GA_12_ and GA_53_ (Schomburg et al., 2003). Distinction between Class I and Class II enzymes has been based on amino acid sequence and catalytic activity (Thomas et al., 1999; Ubeda-Tomás et al., 2006; Serrani et al., 2007). It is estimated that C19-GA2ox evolved prior to the establishment of gymnosperms followed by the appearance of C20-GA2ox (Class III) and subclassification of C19-GA2ox into Classes I and II before the emergence of angiosperms, with gene copy number within each class expanding in further duplication events (Huang et al., 2015; Yoshida et al., 2020; Hernández-García et al., 2021). In Arabidopsis, Class I enzymes include GA2ox1, GA2ox2, and GA2ox3, whereas Class II enzymes include GA2ox4 and GA2ox6 (*GA2ox5* is a pseudogene). Class III comprises GA2ox7, GA2ox8 and the recently characterized GA2ox9 and GA2ox10 (Lange et al., 2020).

By their ability to reduce or eliminate bioactive GAs, the GA2ox enzymes have potential to govern the domains of bioactive GAs, and thus may have a paramount role in influencing patterns of GA trafficking and GA-dependent processes. Previous research has identified diverse roles for *GA2ox* genes in various plants. For example, in both rice and Arabidopsis, localized expression of specific *GA2ox* genes at the base of the meristem isolates the meristem from surrounding GA-rich tissues (Sakamoto et al., 2001; Jasinski et al., 2005). In Arabidopsis, touch-induced expression of the *AtGA2ox7* gene mediates GA-dependent thigmomorphogenesis (Lange and Lange, 2015), while induction of the same gene under salt stress represses growth, potentially to allow for better survival (Magome et al., 2008). Thus, understanding the genomic complement of the *GA2ox* genes, and when and where the individual genes are expressed, should give new insight into functions of GAs in woody plant growth, development and physiology, including stress responses.

In apple (*Malus x domestica* Borkh.), one of the most widely grown and economically important temperate tree fruits, GAs influence numerous aspects of development and physiology that condition production traits important for yield, fruit quality and sustainability of production. A notable example is floral induction. In apple, as well as many other tree fruit species, exogenous GAs generally have a repressive effect on floral induction (Tromp, 1982; Southwick et al., 1995; Bertelsen and Tustin, 2002; Goldberg-Moeller et al., 2013; Zhang et al., 2016; Zhang et al., 2019). In addition, in many important apple cultivars, GAs produced in the seeds of developing fruit have been thought to repress floral initiation on adjacent bourse shoots, leading to reduced flowering and fruiting the following season, a major production problem termed alternate or biennial bearing (Luckwill et al., 1969). Despite its fundamental and applied importance, relatively little is known about the components of GA signaling in apple and their relationships with flowering.

Previously, we identified four *MdGA2ox* genes, now named as *MdGA2ox1A*, *-1B*, *-2A* and *-2B*, that were rapidly and persistently induced in the apple shoot apex in response to exogenous GA_4+7_ as well as by the presence of fruits during the anticipated period of floral induction (Zhang et al., 2019; Gottschalk et al., 2021). These results reflect a feedforward GA dynamic and prompted us to initiate the development of *MdGA2ox* genes as models to study early events in GA signaling and the potential roles of *GA2ox* genes in growth and development of apple and other perennial tree species. In the current study, we identified canonical GA2ox-like genes in the apple genome, documented their genomic organization and homeology, and assessed potential functional redundancy or divergence by identifying their developmental, diurnal, and stress-related expression profiles. This study provides a biological and evolutionary perspective into GA2ox functions in apple with an emphasis on floral induction.

## 2 Materials and methods

### 2.1 Genome-wide census of apple *GA2ox* genes

Homology-based identification of *MdGA2ox* genes among all genes previously annotated in the GDDH13 reference genome used the BLAST (Basic Local Alignment Search Tool) algorithm (Altschul et al., 1990) and the GDDH13 protein (https://iris.angers.inra.fr/gddh13/downloads/GDDH13_1-1_prot.fasta), or mRNA (https://iris.angers.inra.fr/gddh13/downloads/GDDH13_1-1_mrna.fasta) reference datasets. The query sequences comprised all 14 peptide sequences cataloged as “Gibberellin 2-beta-dioxygenase” in ExPASy (https://enzyme.expasy.org/EC/1.14.11.13): 7 GA2ox sequences from Arabidopsis, 4 from rice, 2 from pea, and 1 from bean (UniProtKB/Swiss-Prot accessions: G2OX1_ARATH, Q8LEA2; G2OX2_ARATH, Q9XFR9; G2OX3_ARATH, O64692; G2OX4_ARATH, Q9C7Z1; G2OX6_ARATH, Q9FZ21; G2OX7_ARATH, Q9C6I4; G2OX8_ARATH, O49561; G2OX1_ORYSJ, Q5W726; G2OX2_ORYSJ, Q5ZA21; G2OX3_ORYSJ, Q8S0S6; G2OX6_ORYSJ, Q7XP65; G2OX1_PEA, Q9SQ80; G2OX2_PEA, Q9XHM5; G2OX_PHACN, Q9XG83) (**Supplementary File 1**). At an Expect (e)-value cutoff of 1E-12, this resulted in the identification of 185 GDDH13 gene names. The designated open reading frame translation from each gene model was retrieved from the GDDH13 protein database (https://iris.angers.inra.fr/gddh13/downloads/GDDH13_1-1_prot.fasta) (**Supplementary File 2**) and used as a query to reciprocally interrogate a dataset of Arabidopsis open reading frame translations (TAIR10_pep_20101214; TAIR10; http://arabidopsis.org) with e<1E-12. This resulted in 118 Arabidopsis protein names (**Supplementary File 3**).

HMMer (http://hmmer.org) was implemented with the hmmbuild module to create a profile based on the multiple alignment of the 14 GA2ox index sequences. The profile was then used as a query with hmmsearch and an e-value of 0.01 for both whole sequence and best match domain. This approach resulted in identification of 233 apple genes. Protein sequences of these 233 homologs were retrieved from the reference genome (GDDH13_1-1_prot.fasta) using shell scripts. These sequences were scanned for domains in the Pfam database (El-Gebali et al., 2019; https://pfam.xfam.org).

**Figure 1:**
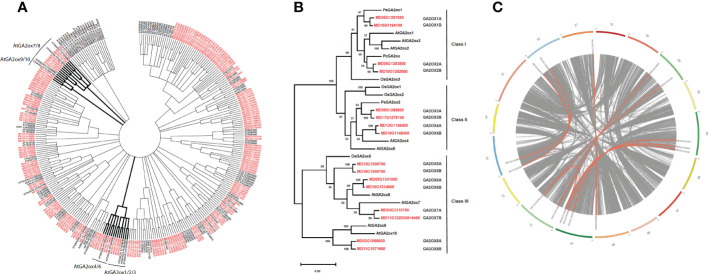
Phylogenetic analyses of GA2ox-related protein sequences. **(A)** Phylogenetic analyses of GA2ox-related protein sequences identified by BLASTp. Apple and Arabidopsis sequences are shown in red and black font, respectively. Clades containing canonical GA2ox representatives from Arabidopsis are shown in bold. A bootstrap cutoff value of 70 was used to define branches. **(B)** Phylogenetic tree of apple GA2ox protein sequences (red font) and characterized GA2ox proteins from Arabidopsis, rice, pea and bean. **(C)** Circos plot showing chromosomal position of the *MdGA2ox* genes with respect to homeologous chromosomes, which are designated with similar color. Homeologous genes are connected with an orange line.

As HMMer analysis did not result in identification of additional homologs, only the first set of sequences along with 14 query sequences were aligned separately, using MUSCLE (Edgar, 2004) and then analyzed for phylogenetic relationship using MEGAX (version 10.1.8) with the phylogenetic tree shown in **Figures 1A, B** generated using the maximum likelihood method and a bootstrap of 1000 replicates. All positions with less than 80% site coverage were eliminated (partial deletion option). There were a total of 306 positions in the final dataset.

### 2.2 Gene, transcript and protein model analyses

Gene models were based on the GDDH13 annotation (Daccord et al., 2017) and curated with the ‘Gala’ phased diploid genome (Sun et al., 2020); http://bioinfo.bti.cornell.edu/apple_genome/) as well as transcriptome datasets derived from the ‘Gala’ shoot apex (Zhang et al., 2019; NCBI Sequence Read Archive PRJNA299491). Gene models were visualized using IGV (version 2.8.13; Robinson et al., 2011) and SnapGene^®^ software (version 5.3.2; from Insightful Science; available at snapgene.com). As the ‘Gala’ reference genome is more consistent with our transcriptome datasets in general, the sequences of the consensus gene models were used for protein structure analyses and to build the refined phylogenetic tree shown in **Figure 1B**. Loci with inconsistent gene models were curated based on the reads from the transcriptome datasets. Putative amino acid sequences were predicted based on the open reading frame sequences using ORF Finder (https://www.bioinformatics.org/sms2/orf_find.htm; Stothard, 2000). Protein structures from novel transcripts and curated gene models were analyzed using SMART (Simple Molecular Architecture Research Tool, http://smart.embl-heidelberg.de/; Letunic and Bork, 2018).

### 2.3 Plant materials and growth conditions

‘Gala’ apple trees (‘Brookfield Gala’ grafted on ‘Pajam 2’ rootstocks) used to detect the expression of *MdGA2ox* genes in response to GA and in spur structures were located at the Michigan State University Research Center in Clarksville, Michigan (42°52’24”N, 85°15’30”W) and were managed in accordance with standard commercial practices for disease, insect and weed control. The date of full bloom was defined as the date on which the maximum numbers of flowers were at anthesis. Three trees were sprayed with GA_3_ at a concentration of 400 parts per million (ppm) at 30 days after full bloom (DAFB); three control trees were sprayed with water; each tree served as a biological replicate. Regulaid (0.1%) (Kalo, Overland Park, Kansas) was used as surfactant for each spray. For gene expression in spur structures, there were three biological replicates with each consisting of 15 spurs from three trees (5 spurs/tree). Fruiting spurs were dissected into 11 structures including shoot apex, bourse leaf, petiole of the bourse leaf, base of bourse leaf petiole, spur leaf, petiole of spur leaf, base of spur leaf petiole, pedicel, base of pedicel, immature seeds and fruit (minus seeds; longitudinally cut with skin, ~3 mm thick). Transverse sections (~2 mm thick) of the fully expanded bourse leaf and spur leaf that were most adjacent to the shoot apex and the largest fruit, respectively, were dissected. Non-fruiting spurs were dissected into 4 structures, including shoot apex, bourse leaf, petiole of bourse leaf and base of bourse leaf petiole. Samples were collected at 40 DAFB and placed immediately into liquid nitrogen and stored at -80°C until use. For seed structures, fruit was collected at 37 DAFB and embryo, endosperm and seed coat were dissected from about 150 immature seeds per biological replicate. Three trees served as three biological replicates.

For experiments with seedlings, seeds were obtained from fruits of open-pollinated ‘Gala’ trees and subjected to stratification at 4°C for three weeks in moist vermiculite (fruits were pre-stored in the cold for two months). Germinating seeds were transferred into square pots (size: 10 cm X 10 cm X 10 cm) containing artificial soil mix (Sungro Professional Growing Mix, Metro-Mix^®^ 852). Plants were maintained in a controlled environment chamber under 16h-light/8h-dark photoperiods. Lighting was supplied with white fluorescent lights (Philips 32-watt, model: F32T8/TL741, average light intensity: 154 μmol·m^-2^·s^-1^). The temperature was held at 25°C during the light period and 18°C during the dark period. The relative humidity ranged from 25% to 28% in both the light and dark periods. Experiments to evaluate gene expression in various seedling structures, as well as diurnal expression, and changes in expression in response to water loss and salt exposure were carried out three weeks after transplanting, when plants had developed 9-11 true leaves and were ~20 cm tall. For analysis of developmental expression, structures were excised using a razor blade between 5 h and 7 h after the onset of illumination. To evaluate diurnal changes in expression, the first fully expanded leaf and 3-5 mm shoot apex were collected. Each sample comprised pooled tissue from 10-15 seedlings, and all experiments utilized three biological replicates sampled within 5 min. Dissected tissue was immediately frozen in liquid N_2_.

### 2.4 Leaf water loss experiment

To evaluate gene expression in the leaf associated with water loss, the first fully expanded leaf was excised at the base of the petiole using a razor blade and placed on a paper towel at ambient conditions. Specifically, a total of 54 plants were randomly assigned into two groups (air drying and control). Each group consisted of eight subgroups. Each subgroup had three replicates with each replicate consisting of a total of three leaves taken from three individual plants. The leaf was weighed immediately following the cut. The “air drying” group of leaves were left on the bench in the same growth room with no change in the environmental conditions. For the control, leaves were kept hydrated by putting the petioles in water. Eight subgroups of leaves in both “air drying” and control were weighed again and then collected into liquid nitrogen for RNA extraction at 10 min, 30 min, 2 h, and 6 h. The beginning of the treatment was 5 h to 6 h after the onset of illumination.

### 2.5 Salt treatment

To examine the expression of *MdGA2ox* genes in response to salt, ~500 seedlings were well-watered and fertilized two days before the experiment. About 2 h prior to treatments, the plants were watered accordingly to ensure that the soil in each pot was fully saturated with water, and excessive water in the bottom tray was drained before treatments. The plants were then irrigated with 100 mL of 100 mM NaCl or 100 mL of water (control). Excess salt solution or water in the bottom tray was drained immediately following the treatment. Young leaves with a diameter of 0.5-1 cm (one leaf per seedling) were excised at 10 min, 30 min, 2 h, 6 h, 1 d, 2 d, and 6 d after treatment, respectively, and quickly collected into liquid nitrogen. There were three biological replicates at each time point in two treatment groups (salt or control) with each replicate containing 10-15 young leaves.

### 2.6 RNA extraction and RT-PCR

Total RNA was extracted using the method of Gasic et al. (2004) with the exception that spermine was substituted for spermidine in the extraction buffer, followed by a final ‘clean-up’ step using a commercial kit (RNeasy Mini; Qiagen). RNA was treated with DNase I (Qiagen) to remove genomic DNA. A small amount of RNA was checked on a Nanodrop spectrophotometer and an agarose gel for quantity, quality, and integrity. About 1 ug of RNA was then reverse-transcribed to cDNA using the High-Capacity Reverse Transcriptase Kit (ThermoFisher). Taqman primer and probe sets were designed according to the curated *MdGA2ox* transcript sequences in our ‘Gala’ dataset. The primer/probe design rules and quantitative RT-PCR conditions were as described in our previous study (Zhang et al., 2019).

### 2.7 Cluster and correlation analysis

Clustering and correlation analysis was conducted in RStudio (version 1.1.463) using packages Hmisc and corrplot, respectively. Euclidean distance followed by implementation of the Ward 2 algorithm was used for clustering, while Pearson correlation coefficients were used for correlation analysis.

## 3 Results

### 3.1 Census and genomic organization of apple *GA2ox* genes

As a first step to comprehensively identify canonical *GA2ox* genes in apple, we carried out a census of GA2ox genes as annotated for the genome of a doubled-haploid derivative of cv. ‘Golden Delicious’ (‘GDDH13’; Daccord et al., 2017). The protein BLAST sequence homology search tool (Altschul et al., 1990) was used with 14 protein sequences from Arabidopsis, *Oryza sativa* (rice), *Pisum sativum* (pea) or *Phaseolus coccineus* (runner bean) cataloged as “Gibberellin 2-beta-dioxygenase” in the ExPASy Enzyme database as queries (**Supplementary File 1**). All of the 185 apple sequences showing significant homology (**Supplementary File 2**) were reciprocally used as queries to identify more distantly related sequences (118) from Arabidopsis (**Supplementary File 3**). We additionally used a Hidden-Markov-Model-based approach (HMMer; Johnson et al., 2010) with sequence motif queries derived from the conserved domains of the 14 cataloged GA2ox proteins. However, HMMer analysis did not result in identification of additional homologs (**Supplementary File 4**). All identified sequences from both apple and Arabidopsis were subjected to phylogenetic analyses (**Figure 1A**).

We found that the known Arabidopsis GA2ox sequences were represented by three clades, one containing AtGA2ox1-AtGA2ox4 and AtGA2ox6 along with eight apple sequences, one containing AtGA2ox7 and AtGA2ox8 and seven apple sequences, and one containing AtGA2ox9 and AtGA2ox10 along with two apple sequences. The AtGA2ox1/2/3/4/6 clade was further defined by two subclades, one containing AtGA2ox1-3 and four apple sequences, and the other containing AtGA2ox4 and AtGA2ox6 and four apple sequences (**Figure 1A**). The 17 apple sequences included in these three clades were also those that showed the strongest homology with each of the individual GA2ox query sequences, as expected. The next highest scoring apple sequences identified with BLAST were MD13G1170400, whose closest Arabidopsis homolog is LBO1 (LATERAL BRANCHING OXIDOREDUCTASE 1), and MD07G1299900 and MD01G1228800, both most closely related to Arabidopsis DMR6 (DOWNY MILDEW RESISTANCE 6) (**Figure 1A**). These collective results suggested that the GDDH13 reference genome annotation included 17 canonical GA2ox sequences.

**Figure 2:**
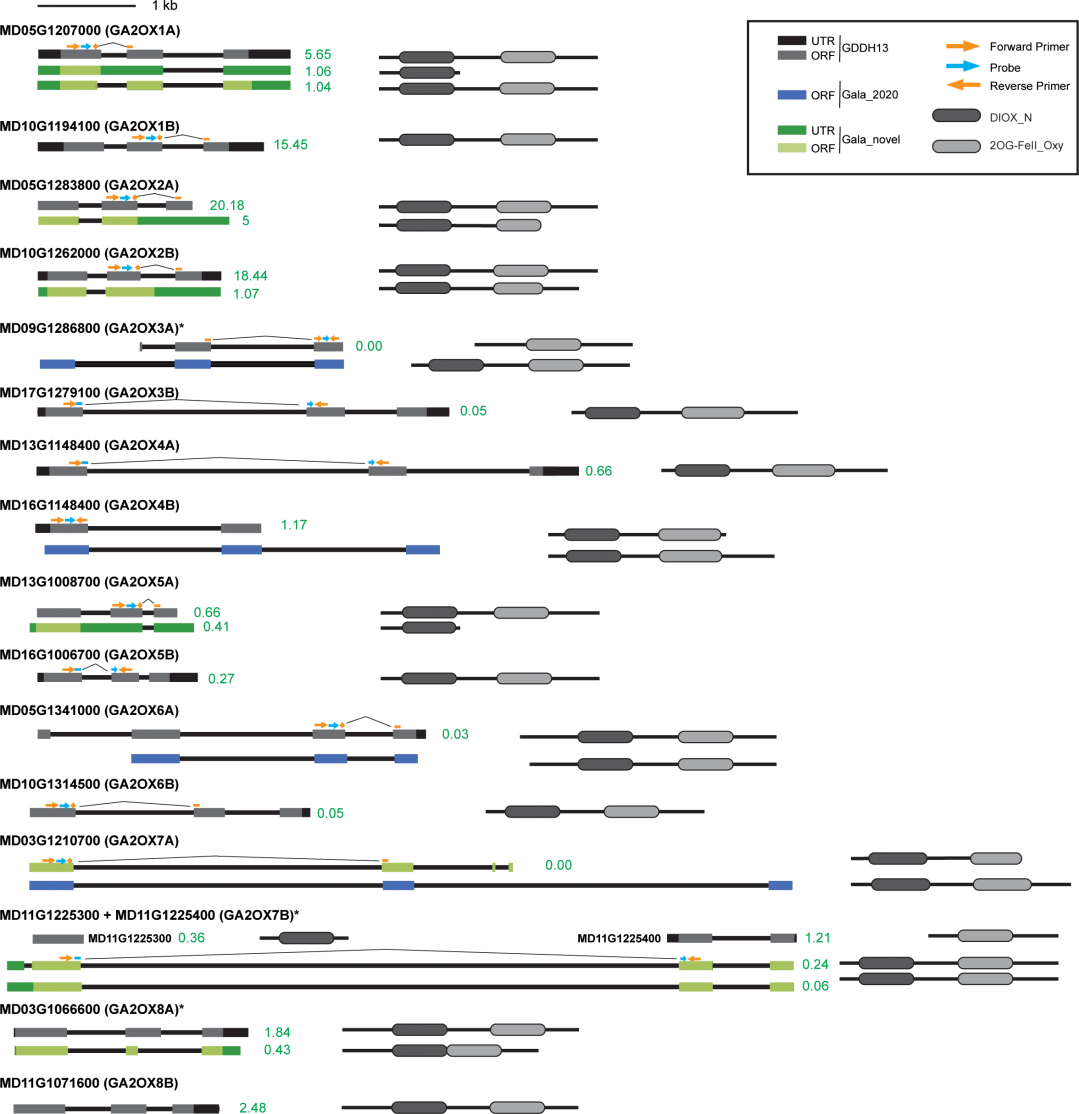
*MdGA2ox* gene, transcript, and protein models. Primary gene models as annotated for the GDDH13 genome are shown at left in black/gray. The gene models annotated in the ‘Gala’ genome are mostly consistent with the ‘GDDH13’ genome, except for four loci (*MdGA2ox3A*, *-4B*, *-6A* and *-7A*) where ‘Gala’ models are depicted in blue. Additional transcript models from ‘Gala’ based on our data are shown in green. Untranslated regions (UTRs) (black or dark green) and exons (gray, light green or blue) are indicated as boxes. A scale bar for transcript models is given at the top. Positions of Taqman primers (orange) and probes (light blue) are shown as arrows. Estimates of expression levels as FPKM (Fragments Per Kilobase of transcript per Million mapped reads) for each transcript in the ‘Gala’ shoot apex are shown in green next to the transcript models.

To evaluate this annotation of the GDDH13 genome, transcript models for the 17 genes were generated using data from our RNA-seq-based profiling of the shoot apex of ‘Gala’ (Zhang et al., 2019) and mapping to both GDDH13 and a recently released, phased-diploid ‘Gala’ genome sequence (Sun et al., 2020) (**Supplementary Files 5** and **Supplementary File 6**, respectively). GDDH13 and Gala annotations were generally consistent with our results, with a few exceptions (**Figure 2**). First, the ‘Gala’ genome supports a more extensive and distinct first exon of *MD09G1286800*. Second, the ‘Gala’ genome and our transcriptome data indicate that the transcribed region of *MD16G1148400* is more extensive and contains a third exon. Third, the ‘Gala’ genome annotates three exons in the transcribed region of *MD05G1341000*, rather than four as annotated in the GDDH13 genome. Fourth, the ‘Gala’ genome supports a more extensive transcribed region as well as a distinct third exon for *MD03G1210700*. Finally, we identified a single transcript model including both *MD11G1225300* and *MD11G1225400*. We verified that these two distinctly annotated loci generate a common transcript with an atypically long (~5.9 kbp) intron using a Taqman PCR primer-probe set (**Figure 2** and **Supplementary File 7**). Additional transcript models were identified in the transcriptome datasets for several of the MdGA2ox genes. Most of these represented alternatively spliced forms with premature termination codons within the open reading frames (**Figure 2**).

Canonical GA2ox proteins comprise two highly conserved peptide sequences: an amino-terminal segment found within proteins with 2-oxoglutarate/Fe(II)-dependent dioxygenase activity (DIOX_N) and a carboxyl-terminal segment that define members of the 2OG-Fe(II) oxygenase superfamily (2OG-FEII_Oxy). A total of 20 transcript models, representing 16 genes, could encode a protein containing both domains (**Figure 2** and **Supplementary File 4**). Two genes, *MD05G1207000* and *MD13G1008700*, produced detectable levels of apparently unspliced transcripts in which premature translation termination would lead to loss of the 2OG-FEII_Oxy domain. The DIOX_N domain was absent in the transcript model for *MD09G1286800* annotated in the GDDH13 genome, however, both domains were present according to the gene model annotated in the ‘Gala’ genome. The tandem reference genes *MD11G1225300* and *MD11G1225400* encode a DIOX_N domain and 2OG-FEII_Oxy domain, respectively, further supporting that these loci represent one gene. The 2OG-FEII_Oxy domain comprises the catalytic core that interacts with 2-oxoglutarate (including the conserved residues His, Asp, and His) and Fe^2+^ (Arg, Ser) (Huang et al., 2015). All of the major gene models contain these conserved residues at the expected positions (**Supplementary File 8**).

Based on this data, we concluded that there are a total of 16 canonical *GA2ox*-like genes in these apple genomes. A refined phylogenetic tree of apple *GA2ox* genes and selected relatives from other plants is shown in **Figure 1B**. This phylogeny suggested that these genes represent eight pairs of duplicated genes, and this idea is consistent with their annotated chromosomal locations within the apple genome and syntenic relationship among apple chromosomes (**Figure 1C**) (Daccord et al., 2017). These apple *GA2ox* genes were named based on their phylogenetic relationship and genomic organization. Four (*MdGA2ox1A*/*1B* and *-2A*/*2B*) are clustered in Class I, four (*MdGA2ox3A*/*3B* and *-4A*/*4B*) in Class II, and eight (*MdGA2ox5A*/*5B*, *-6A*/*6B*, *-7A*/*7B*, and *-8A*/*8B*) in Class III (**Figure 1B**). The characterization of the transcriptional activity of the genes described below omitted *MdGA2ox8A/8B*, the apple homologs of Arabidopsis *GA2ox9* and *GA2ox10*, as these Arabidopsis genes were not recognized as *GA2ox* genes when our experiments were initiated.

### 3.2 GA feedforward regulation of apple *GA2ox* genes

**Figure 3:**
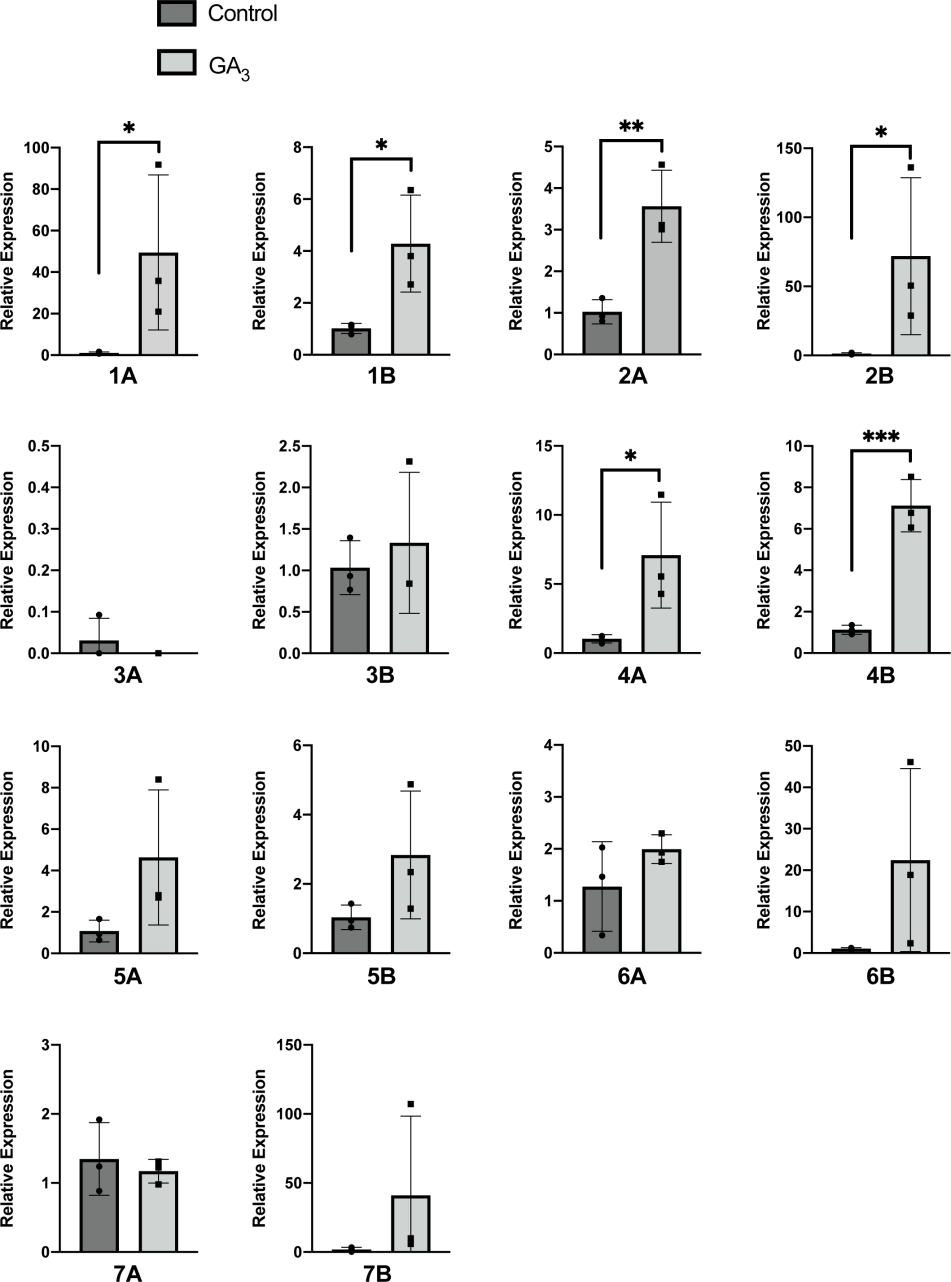
Expression of *MdGA2ox* genes in the apple shoot apex in response to applied GA_3_. ‘Gala’ trees were subjected to a foliar application of either 400 ppm of GA_3_ or water (control) at 30 DAFB, and the shoot apices were collected 2 d after treatment. Black dots represent individual values from three biological replicates. Relative expression was calculated based on the expression ratio between *GA2ox* and a reference *MdACTIN* gene. An error bar indicates standard deviation among the three biological replicates, while asterisks denote statistical significance (*, p < 0.05; **, p < 0.01, ***, p < 0.001).

Our previous finding that the expression of at least four *MdGA2ox g*enes was rapidly increased in the apple shoot apex after exposure to GA_4+7_ suggests a feed-forward mechanism. In the present study, we examined the transcriptional responses of the *GA2ox* genes in the shoot apex, two days following a foliar-applied commercial formulation of GA_3_ at 30 days after full bloom (DAFB) **(** **Figure 3** **)**. Most of the genes, including the four previously identified as GA-responsive (*MdGA2ox1A/1B*, and -*2A*/*2B*), were expressed to higher levels in the GA-treated plants. This upregulation by GA at this stage was most striking for the Class I/II *MdGA2ox1A*/*1B*, *-2A*/*2B*, and *-4A*/*4B* (**Figure 3**). Four genes (*MdGA2ox3A/3B*, *-6A* and *-7A*) did not show obvious upregulation by the applied GA.

### 3.3 Developmental expression pattern of apple *GA2ox* genes

**Figure 4:**
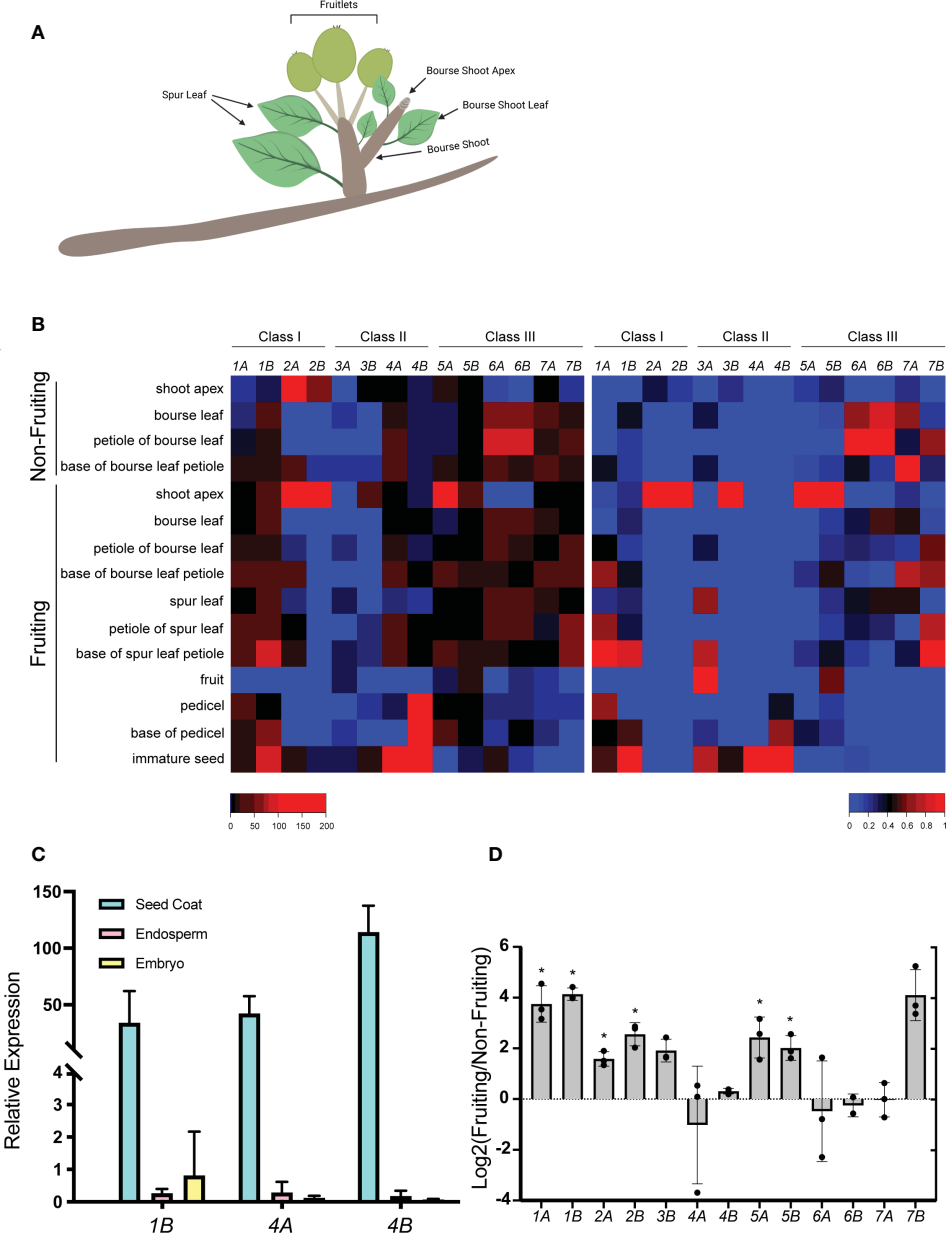
Expression profiles of *MdGA2ox* genes in spur and seed structures. **(A)**. Illustration of a fruiting apple spur at the time of analysis. **(B)**. Expression of *MdGA2ox* genes in structures dissected from non-fruiting or fruiting spurs. The heat map on the left represents expression values derived from quantitative PCR and relative to an apple *ACTIN* gene. The heat map on the right represents expression values relative to the strongest expression for each gene among structures. Color keys at the bottom indicate expression levels from low/least (blue) to high/greatest (red). **(C)**. Relative expression of *MdGA2ox1B*, *-4A*, and *-4B* in the seed coat, endosperm, and embryo of the developing seed. **(D)**. Relative *MdGA2ox* expression in fruiting and non-fruiting shoot apex. Log_2_(Fruiting/Non-fruiting) represents the fold difference of relative expression in the shoot apex of fruiting spurs versus that of non-fruiting spurs. Black dots indicate individual values of three biological replicates, and error bars represent standard deviation among replicates. Asterisks indicate statistical significance (t-test, p < 0.05).

To evaluate developmental regulation of the *MdGA2ox* genes, their expression was monitored within 11 structures of fruiting spurs at 40 DAFB (**Figure 4**). Spurs are condensed shoots comprising structures initiated during the previous season (spur leaves, flowers/fruit) and structures initiated in the current season (bourse shoot and floral primordia (**Figure 4A**). Unlike many cultivars, ‘Gala’ typically forms flowers on bourse shoots irrespective of the presence of developing fruit (Zhang et al., 2019), and so the bourse shoot apices analyzed here were assumed to be committed to flowering.

Nearly all of the structures analyzed showed expression of multiple *MdGA2ox* genes, with one or a few genes predominating (**Figure 4B**). The petiole of both spur and bourse leaves, especially the dissected petiole base, showed the greatest number of distinct genes expressed, whereas in the fruit (whole fruit minus seed) only one (*MdGA2ox5B*) was appreciably expressed (**Figure 4B**). *MdGA2ox2A/2B* and *-5A* dominated expression in the shoot apex; *-1B* and *-6A/6B* in the bourse and spur leaf; *-6A* and *-7B* in the bourse leaf petiole, *-1B* in the base of the spur leaf petiole, *-4B* in the pedicel, and *-1B* and *-4A/4B* in the immature seed. Each *GA2ox* gene also showed strong structure-specific expression (**Figure 4B**): *MdGA2ox1A* was preferentially expressed in the petiole, especially the base; *-1B* in the petiole base and immature seed; *-2A/2B*, *-3B*, and *-5A/5B* in the shoot apex; *-3A* in the fruit; *-4A* and *-4B* in the immature seed; and *-7B* in the base of the spur leaf petiole. For the three genes that showed strongest expression in the immature seed - *MdGA2ox1B*, -*4A*, and *-4B*, - expression was further analyzed within the seed coat, endosperm and embryo (**Figure 4C**) at 33 DAFB, when the embryo was patterned but prior to enlargement and desiccation. This revealed that the expression of these three genes was largely confined to the seed coat (**Figure 4C**).

It has been hypothesized that developing fruit comprise a source of GAs that can repress floral initiation on the proximal bourse shoot. To assess the potential effect of developing fruit, and potentially GAs, on the expression of the 14 *MdGA2ox* genes, their expression was also monitored within non-fruiting spur structures (**Figure 4B**). We noted several cases in which individual *MdGA2ox* genes were expressed differentially in the same structure between fruiting and non-fruiting spurs. Most strikingly, in the shoot apex, six genes, comprising three homeologous pairs (*MdGA2ox1A/1B*, *-2A/-2B*, and *-5A/5B*), were expressed to significantly higher levels in the presence of fruit (**Figure 4D**). In contrast, *MdGA2ox6A* and *-6B* were expressed highly in the bourse leaf and its petiole in non-fruiting spurs, but not in fruiting spurs (**Figure 4B**). It is worth noting that *MdGA2ox1A*/*1B* and *-2A/-2B* were apparently responsive to both GA and fruit, whereas *MdGA2ox4A*/-*4B* were responsive to GA but not fruit, and *MdGA2ox5A/-5B* were responsive to fruit but not GA (**Figures 3**, **Figure 4**). The relative expression levels of these *MdGA2ox* genes estimated from qRT-PCR correlated well with direct counting of expressed-gene fragments, at least for the shoot apex, where RNA-seq data was available (**Figure 2**).

**Figure 5:**
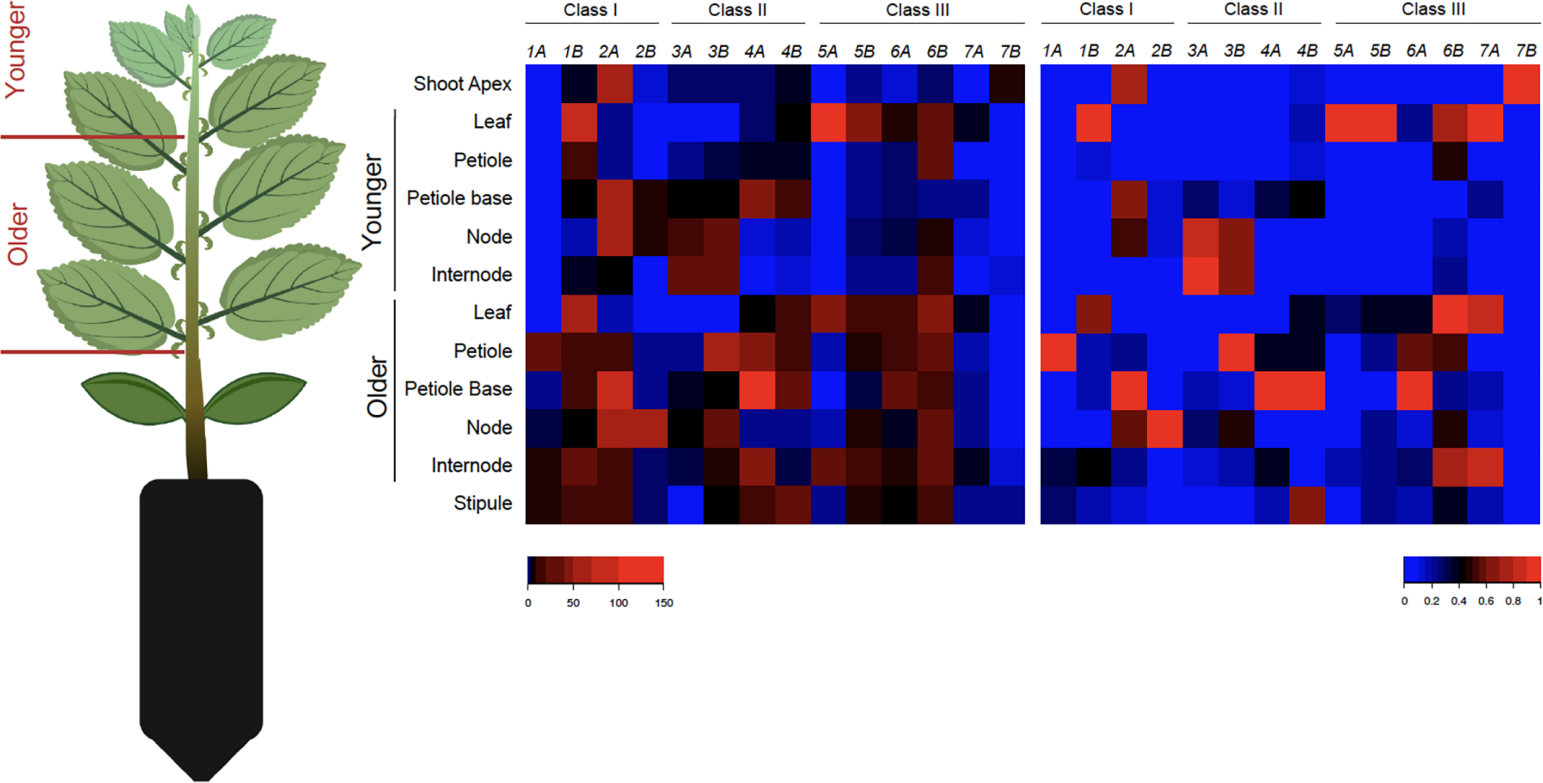
Expression profiles of *MdGA2ox* genes in apple seedling structures. Seedlings were partitioned into two general sections, Younger, comprising all structures apical to the most apical fully expanded leaf and Older, comprising this leaf and all basipetal structures. Younger and older stipules were pooled. The heat map on the left represents expression values derived from quantitative PCR and relative to an apple *ACTIN* gene. The heat map on the right represents expression values relative to the strongest expression for each gene among structures. Color keys at the bottom indicate expression levels from low/least (blue) to high/greatest (red).

Expression of *MdGA2ox* genes was also evaluated in various structures dissected from rapidly growing seedlings that were ~6 weeks old and had produced 9-11 true leaves. These structures included the shoot apex (containing leaf primordia and leaves <5 mm in length), young structures taken from the apical section of the seedling (leaf, petiole, base of the petiole, stem node and internode) and the same structures from the older, central section of the seedling (leaf, petiole, base of the petiole, stem node and internode). In addition, stipules pooled from the younger and older sections were analyzed. Similar to the spur, nearly all of the structures analyzed showed expression of multiple *MdGA2ox* genes, with one or a few genes predominating (**Figure 5**). In the petiole and its base of older leaves, as well as the internode of the older stem, and stipule, the majority of the genes were strongly expressed, whereas in the shoot apex only *MdGA2ox2A* was strongly expressed. *MdGA2ox1B*, *-5A/B* and *-6B* dominated expression in the leaf, *MdGA2ox2A* and *-4A* in the base of the petiole, and *MdGA2ox2A* in the node. Also similar to the spur, each *MdGA2ox* gene also showed strong structure-specific expression (**Figure 5**). *MdGA2ox1A* and *-3B* were preferentially expressed in the petiole of older leaves, *-1B*, *-5A* and *-5B* in the young leaf, -*2A*, *-4A/B* and *-6A* in the base of the older leaf petiole; *-2B* in the older stem node, *-3A/B* in the young node and internode; *-3B* also in the older petiole, *-6B* and *-7A* in the leaf and in the internode of older stem; and *-7B* in the shoot apex.

Expression levels of individual genes were compared between the structures from the growing, apical section of the seedlings and the fully expanded, more basal section. In the leaf, nearly all genes were expressed to similar levels in younger and older structures. In contrast, in the petiole, base of the petiole, node, and internode, several genes were expressed to higher levels in the older structures: *MdGA2ox1A*, *-2A*, *-3B*, *-4A/B* and *-6A* in the petiole; *-1B*, *-2A*, *-4A/B*, and *-6A* in the petiole base; *-2B*, *-3B*, *-5B* and *-6B* in the node; and *-1A/B*, *-2A*, *-4A*, *-5A/B*, and *-6B* in the internode. Three of the genes, *MdGA2ox1A*, *-2B*, and *-4A*, were expressed almost exclusively only in the older structures (**Figure 5**).

### 3.4 Diurnal changes in expression

**Figure 6:**
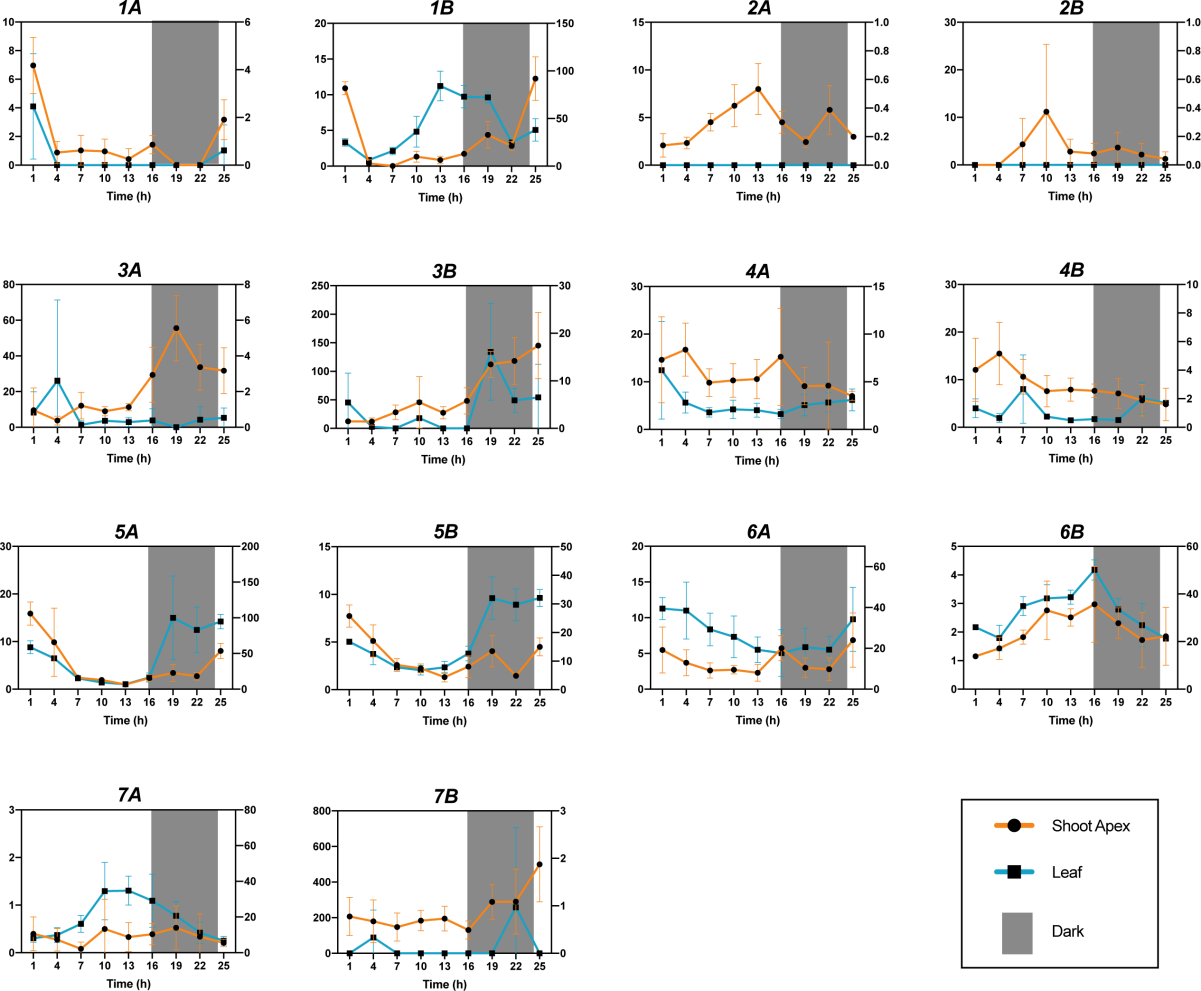
Diurnal expression of *MdGA2ox* genes in the shoot apex and first fully expanded leaf in seedlings. The shoot apex and first fully expanded leaf were excised from rapidly growing seedlings maintained under 16-h light/8-hr dark photoperiods. The y axis represents relative expression. Relative expression in the shoot apex and leaf is represented with orange and blue lines, respectively. The grey box in each graph indicates the period of darkness.

To better interpret the developmental expression patterns and guide further analyses of gene expression over time, diurnal expression of each of the 14 genes was examined over one diurnal cycle. Expression was evaluated every 3 h in the shoot apex and most apical fully expanded leaf of ~6-wk-old seedlings maintained under 16-h light, 8-h dark photoperiods. Nearly all of the genes showed apparently patterned changes in expression over the cycle in both structures (**Figure 6**). However, we were unable to identify a pattern that was consistently shared among the genes. *MdGA2ox1A* and *-1B* showed a convincing decrease in expression at the beginning of the light period, whereas expression of *MdGA2ox5A/5B* and *-6A* decreased gradually throughout the light period. *MdGA2ox1B*, *-2A/2B*, *-6B* and *-7A* showed gradual increase in expression during the light period. Five genes (*MdGA2ox3A/3B*, *-5A/5B*, and *-7B*) showed expression increases at the advent of, or during, the dark period, whereas *MdGA2ox1B*, *-2A*, *-6B*, and *-7A* showed a decrease in expression. These seemed to be structure specific; for example, the strong dark-associated increase in *MdGA2ox3A* expression was observed only in the apex, whereas that of *-5A* and *-5B* was seen only in the leaf (**Figure 6**).

Consistent with the seedling developmental experiments documented in **Figure 5**, each gene generally showed clear preferential expression for the apex or leaf. For example, *MdGA2ox2A*/*2B*, *-3A*/*3B*, and *-7B* had much higher expression levels in the shoot apex than that in the first fully expanded leaf (corresponding to the older leaf in **Figure 5** **)**, whereas *MdGA2ox1B*, *-5A*, *-6B*, and *-7A* were more highly expressed in the leaf. However, interestingly, three genes clearly changed their preferential expression between these structures during the cycle. *MdGA2ox1B* was preferentially expressed in the shoot apex at the beginning of the light period, but in the leaf during the remains of the cycle. *MdGA2ox5A/5B* were also expressed preferentially in the apex early in the cycle, but in the leaf during the dark period (**Figure 6**).

We found only limited similarity between the diurnal expression patterns of homeologous genes. For example, *MdGA2ox1B* but not *-1A* showed a clear increase during the light period, while *MdGA2ox6A*/*6B* showed nearly reciprocal expression patterns throughout the cycle. Additionally, *MdGA2ox7A* but not *-7B* showed a peak of expression midway through the light period (**Figure 6**).

Some expression results were not consistent with simple diurnal oscillation. For example, in the shoot apex both *MdGA2ox3B* and *-7B* were relatively highly expressed at 25h in contrast with 1h, even though these times are diurnally equivalent (**Figure 6**). One possibility to explain this is that the experiment captured a snapshot of increasing expression of these genes during this general stage of seedling development.

### 3.5 Expression in the leaf blade in response to leaf removal

**Figure 7:**
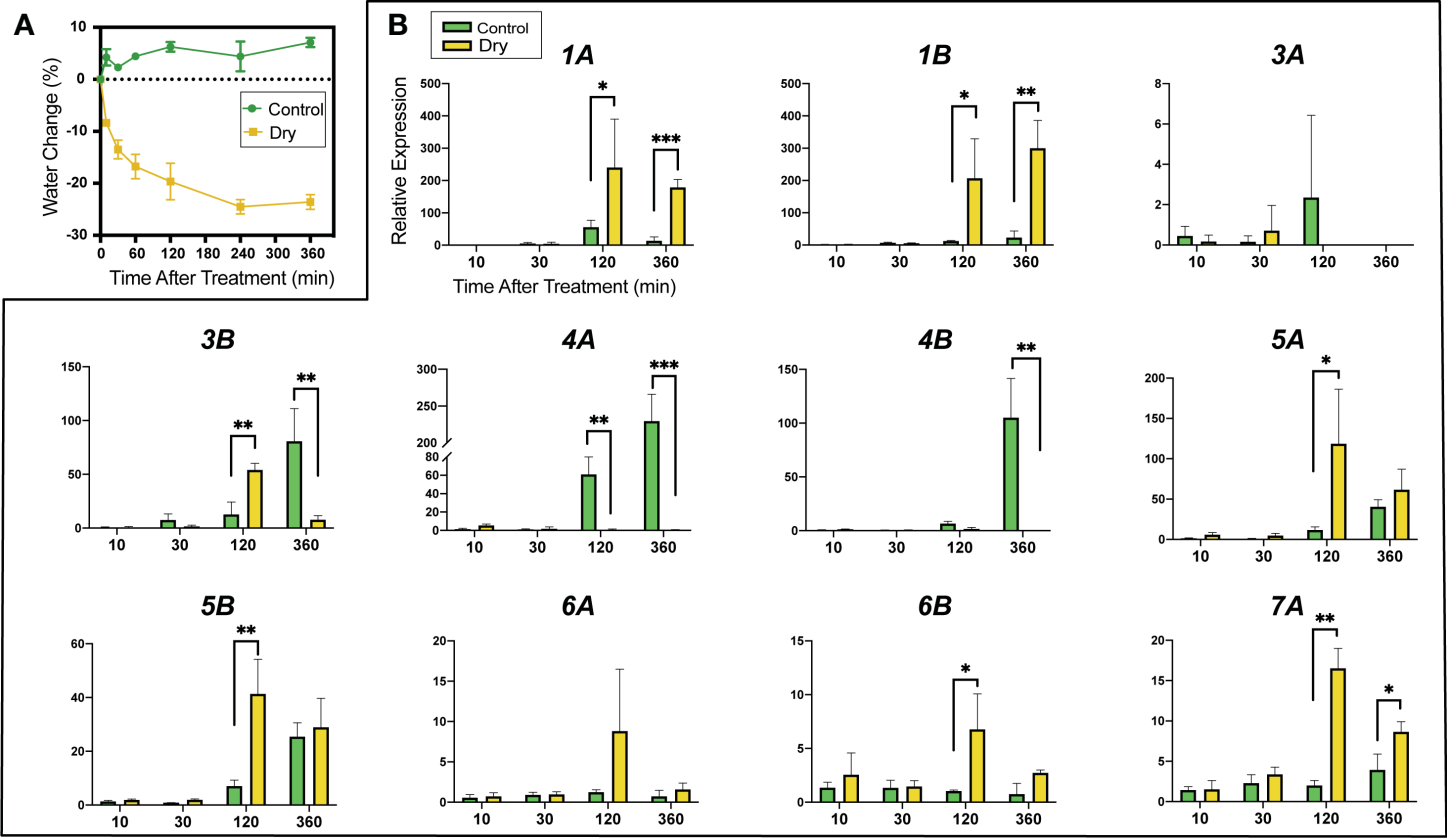
Expression of *MdGA2ox* genes in the leaf blade after leaf excision and induced water loss. The first fully expanded leaf was excised from rapidly growing seedlings and either left on a bench to air dry or kept hydrated with the tip of petiole immersed in water. **(A)**. Changes of water content in the control (green line) and drying (yellow line) leaves. Leaf water change is defined as the difference between the after-treatment weight and the initial weight. **(B)**. Relative gene expression in the control (green bar) and drying (yellow bar) leaves over time. Expression was analyzed at 10 min, 30 min, 120 min (2 h) and 360 min (6 h) after excision. Asterisks denote statistical significance (*, p < 0.05; **, p < 0.01, ***, p < 0.001).

*GA2ox* genes have been identified as important components limiting GA-associated growth during abiotic stress. We evaluated expression of the 14 *MdGA2ox* genes in the blade of the first fully expanded leaf of ~6-wk-old seedlings under conditions of rapid water loss. In this experiment, the leaves were excised and transferred to a paper towel, and allowed to air-dry for up to 6 hr. As a control, leaves were excised and immediately floated in water, with the cut end of the petiole immersed to maintain vascular continuity. Gene expression was assayed at 10, 30, 120, and 360 min after excision. Determination of water content in the samples (**Figure 7A**) indicated that water was lost from the air-drying leaves at least as early as the 10-min time point and further throughout the first 4 h of the treatment.

Expression of three genes, *MdGA2ox2A*/*2B* and *MdGA2ox7B*, was not reliably detected in leaf blades in either the treated or control samples, at any of the four time points. Of the remaining 11 genes, 7 showed increased expression associated with drying (**Figure 7B**), and all of these showed significant induction within 2 h after the beginning of the treatment (**Figure 7B**). This increase was most striking (~200-fold) for *MdGA2ox1A/1B*. In contrast, and unexpectedly, four genes, *MdGA2ox3A/3B*, *-4A* and *-4B*, were expressed to higher levels in the control samples than in the drying samples after 2-6 h. This pattern could not be explained by potential disruption of diurnal cycling by excision, as none of the three genes showed substantial diurnal changes in expression during the six-hour period of the experiment (**Figure 6**).

**Figure 8:**
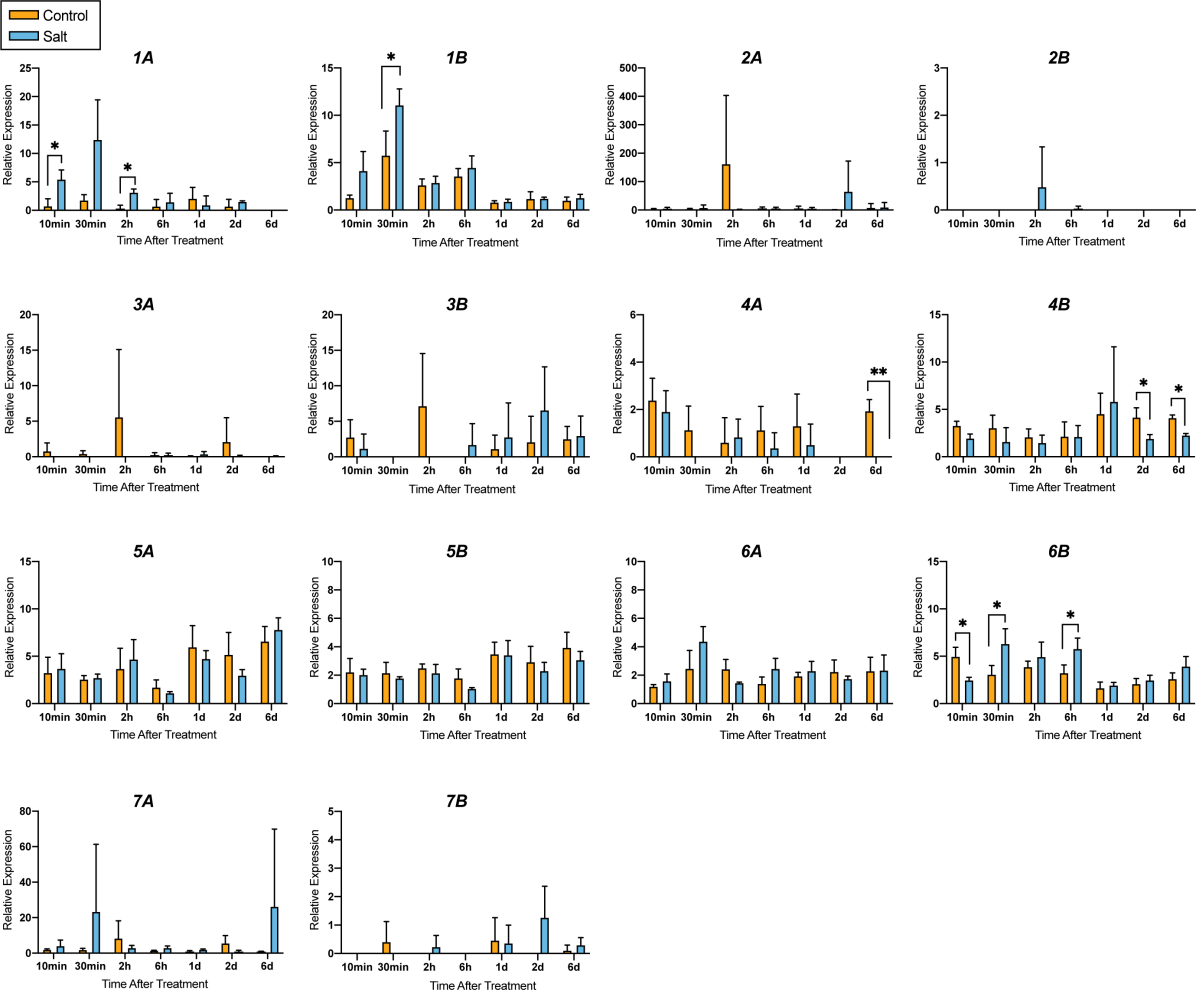
Relative expression of *MdGA2ox* genes in the first fully expanded leaves under salt treatment. Rapidly growing ‘Gala’ apple seedlings were either treated with 100 ml of water (in orange) or with 100 mL of 100 mM salt (NaCl) (in blue). Gene expression was detected at 10 min, 30 min, 2 h, 6 h, 1 d, 2 d, and 6 d after treatment. Asterisks represent statistical significance (*, p < 0.05; **, p < 0.01).

Expression of the 14 genes was also evaluated in response to salt exposure. Six-week-old seedlings growing in soil were subjected to daily watering with 100 mM NaCl, and gene expression was measured in young leaves at 10 min, 30 min, 2 h, 6 h, 1 d, 2 d, and 6 d after the beginning of the treatment. This NaCl concentration was chosen because it was the minimum concentration that noticeably reduced growth in seedlings over a one-week period. Compared with the results of leaf excision, the expression response to salt was subtle (**Figure 8**). *MdGA2ox1A* and *-1B* were induced as early as 10 min after treatment, whereas *MdGA2ox6B* was downregulated at 10 min but upregulated at 30 min and 6 h after treatment. None of these three genes were differentially expressed at later time points (**Figure 8**). Unlike *MdGA2ox1A*, *-1B* or *-6B*, *MdGA2ox4A* and *-4B* were significantly downregulated only at later time points, ~2-6 d after treatment (**Figure 8**).

### 3.6 Cluster and correlation analysis

**Figure 9:**
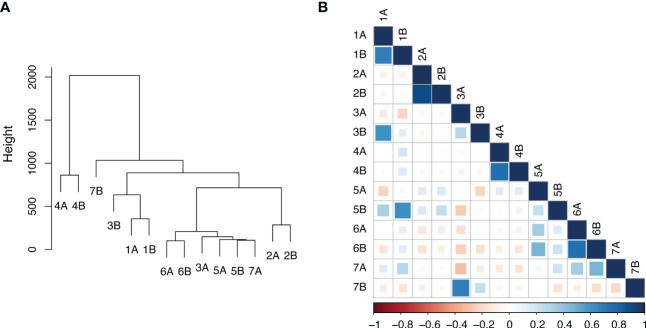
Cluster and correlation analysis of apple *GA2ox* genes. **(A)** The dendrogram depicts the relationship among the 14 *MdGA2ox* genes based on their expression. The y axis represents Euclidean distance (‘Height’). **(B)** Expression correlation. The heat map represents the correlation values between *MdGA2ox* genes (dark blue, strongly positively correlated; dark red, strongly negatively correlated).

To provide an unbiased view of the degree of relationship in expression pattern exhibited by these seven *MdGA2ox* homeologous pairs and individual homeologs, we carried out a clustering analysis of the developmental, diurnal and stress-related experiments described above. *MdGA2ox4A/4B* showed generalized expression characteristics easily distinguishable from the remaining MdGA2ox genes, while several non-homeologous genes fell into the same clade (**Figure 9A**). For homeologs, the strongest correlation was identified between *MdGA2ox2A/2B*, and strong correlation was also found for *MdGA2ox6A/6B*, -*4A/4B* and -*1A/1B*. In contrast, expression of *MdGA2ox7A/7B* was slightly anticorrelated (**Figure 9B**).

## 4 Discussion

Studies to date suggest that GA2ox proteins are key determinants of GA accumulation in plant tissues - Class I/II GA2ox by catabolizing bioactive C19 GAs, and Class III GA2ox by diverting early C20 intermediates from the pathway. Based on our results from two high quality genome sequences and exhaustive transcriptional profiling, coupled with phylogenic analyses, apple contains four members of Class I (MdGA2ox1A/1B, MdGA2ox2A/2B), four of Class II (MdGA2ox3A/3B, MdGA2ox4A/4B), and eight of Class III (MdGA2ox5A/5B, MdGA2ox6A/6B, MdGA2ox7A/7B, MdGA2ox8A/8B). In the absence of post-translational regulation, the expression pattern of the corresponding genes documented here should provide important clues to GA function at the whole-plant level. However, paradoxically, strong expression of *GA2ox* genes within specific domains could represent foci of high GA signaling (for those genes that are GA-responsive), or low bioactive GA concentrations (as a result of GA2ox activity). Obviously, future work defining the substrate specificity of the 16 apple GA2ox, as well as direct measurements of specific GA forms within the shoot apex, will be required to support further hypotheses.

The primary motivation for this study was to identify those *GA2ox* genes that are potentially most important for mediating GA levels in the shoot apical meristem. This is a necessary first step to fully understand how bioactive GAs can repress differentiation of the shoot apex into a floral fate in apple and other woody perennial plants. In the apex of non-fruiting spurs, which typically are undergoing floral initiation, both the direct counting of RNA-seq reads and QRT-PCR analysis suggested that expression was dominated by *MdGA2ox2A/2B*, with at least four additional *MdGA2ox* genes also appreciably expressed. Thus, *MdGA2ox2A/2B* may play a paramount role in governing GA levels in the shoot apex. Interestingly, in the analogous spurs in which developing fruit was present, the expression of several *MdGA2ox* genes was markedly increased, most notably *MdGA2ox1A/1B* and *MdGA2ox5A/5B*. This could reflect increased GA signaling in the apex, although not all of these four genes were significantly induced by exogenous GA_3_. Alternatively, some or all of these genes may be induced through a GA-independent pathway.

It is more intuitive to consider domains of *GA2ox* expression as barriers for the diffusion of GAs from source tissues. A model for this is the localized expression of *OsGA2ox1*, and *AtGA2ox2/AtGA2ox4* at the base of the meristem in the rice and Arabidopsis shoot apex, respectively, which is thought to preclude bioactive leaf-produced GAs from disrupting meristem organization (Sakamoto et al., 2001; Jasinski et al., 2005). In the same way, domains of expression of Class III *GA2ox* could set up a barrier for diffusion of C20 GAs. This idea is supported by the finding that GA_12_ is the major mobile GA signal over long distances in plant tissues (Regnault et al., 2015).

In this context it is notable that our data shows strong *GA2ox* gene expression in the fruit pedicel and leaf petiole. It has long been considered that GAs produced in the seeds of developing fruit diffuse to the shoot meristem to repress floral initiation. In contrast to this idea, we found that several *GA2ox* genes, especially *MdGA2ox4B*, are expressed in the fruit pedicel, suggesting that bioactive GAs produced in the seeds remain isolated. This idea is also strongly supported by the highly localized expression of *MdGA2ox1B -4A*, and *-4B* in the developing seed coat. If expression in the seed coat and/or pedicel is sufficient to metabolize all diffusing seed-produced bioactive GAs, then an established tenet of biennial bearing in apple must be re-examined. In the same way, the observed strong expression of *MdGA2ox6A/6B* in the bourse and spur leaves, including the petiole, may limit GA diffusion from leaves. Supporting this idea in the seedling, several *MdGA2ox* genes expressed in the petiole and petiole base of older leaves were relatively silent in the same structures of younger leaves, suggesting decreased control of GA diffusion. This would be anticipated if leaf-produced GAs were required to assist in promoting internode elongation in the young shoot.

The observation that nearly all of the *MdGA2ox* genes showed at least some indication of diurnal change in expression in rapidly growing seedlings was anticipated, given the pervasive diurnal regulation of genes in plants and the important role for GAs in growth. We hypothesized that bioactive GAs promoting growth of young tissues would be catabolized near or after the advent of darkness, and this would be reflected by increasing *GA2ox* gene expression at the end of the light period. For the leaf, where this should be most apparent, four genes fit this criterion: both *MdGA2ox1B* and *MdGA2ox6B* showed increasing expression throughout most of the light period, whereas *MdGA2ox5A*/*5B* showed strong upregulation at the start of the dark period. Notably, *GA2ox* expression in the young leaf was dominated by these same four genes. Other diurnal patterns are difficult to explain at the whole-structure level, but these could reflect unanticipated operations of GA signaling occurring in limited cell types.

One mechanism by which plants can respond to abiotic stress is the rapid depletion of bioactive GA levels, at least partially through deployment of GA2ox activity, which helps to limit further growth (reviewed in Colebrook et al., 2014). Consistent with previous findings in Arabidopsis (Achard et al., 2008; Magome et al., 2008) we found that subsets of the *MdGA2ox* genes were induced in the leaf blade under conditions of acute water deficit and salt exposure. In excised leaves allowed to air-dry, transcriptional responses were observed in the blade as early as 2 h after excision, and 7 of the 11 genes expressed in the leaf blade showed the expected stress induction. The striking induction of *MdGA2ox1A/1B* was surprising given the already strong expression of these two genes in the blade and petiole, respectively, and suggest that these two genes have an important role in limiting GA-promoted growth.

The unexpected finding that four of the genes were apparently induced when leaves were excised and placed in water is interesting but difficult to explain. Possibly it reflects a response to abrupt loss of negative pressure in the xylem, systemic response to wounding, response to touch (Lange and Lange, 2015) or disconnection from signaling molecules generated outside of the leaf, such as abscisic acid (ABA). These responses may be dysfunctional in the drying leaf due to the dehydration stress. The degree of this response (>100-200-fold induction) suggests that the underlying mechanism is biologically important and worth exploring.

The observation that only a subset of the genes responded significantly to salt, and that these responses were relatively subtle, was surprising, given that the salt concentration used was sufficient to arrest growth. For these genes, increase in expression was seen as early as 10-30 min after watering, suggesting that it reflects a response to decreased osmotic pressure caused by the salt water, rather than by ion toxicity. This is consistent with the observed rapid response of these three genes to leaf drying.

The number of *GA2ox* genes identified in the apple genome is greater than that identified for Arabidopsis (10; Rieu et al., 2008; Lange et al., 2020), maize (13; Li et al., 2021), tomato (11; Chen et al., 2016), grapevine (11; He et al., 2019), poplar (11; Gou et al., 2011), and peach (7; Cheng et al., 2021), and likely reflects not only the depth of our census but also gene family expansion. Neither direct RNA-seq counting for the genes nor the QRT-PCR analyses gave strong indication that individual homeologs had lost all function through silencing of expression. This homeology and retention of homeologs is consistent with the relatively recent whole-genome duplication in apple (Velasco et al., 2010). Retainment of duplicated *GA2ox* gene pairs after whole-genome duplication has been reported in many other flowering plant species and can reflect not only the evolutionary history of the species, but also the utility of the individual homeologs, which is augmented by diversification in function through expression.

Using correlation and clustering analysis based on a small snapshot of expression data, we found that expression of most of the homoeologous pairs was correlated, as expected, indicating potential functional redundancy in specific structures. However, some striking differences in expression patterns between individual homeologs were seen. In the spur, *MdGA2ox4A* but not -*4B* is strongly expressed in the leaf petiole and its base; whereas in the fruit pedicel and its base, *MdGA2ox4A* was essentially silent and *MdGA2ox4B* is strongly expressed. In addition, *MdGA2ox3A* is preferentially expressed in fruit, where *MdGA2ox3B* is silent, while *MdGA2ox3B* is preferentially expressed in the shoot apex, where *MdGA2ox3A* is silent. A final example in the spur is the preferential expression of *MdGA2ox7A* and *-7B* in the base of the bourse or spur leaf petiole, respectively, whereas their homeologs are preferentially expressed in other structures. In the seedling, *MdGA2ox2A* but not -*2B* is strongly expressed in the apex, whereas *MdGA2ox2B* is preferentially expressed in the older node, and *MdGA2ox1B* but not *-1A* is strongly expressed in leaf. Future work is needed to further define divergence in expression patterns among homeologs, and link this with sequence variation within regulatory regions of the genes that may govern this.

Our expression analyses did not include *MdGA2ox8A* and *-8B*, the homeologs of Arabidopsis *AtGA2ox9* and *AtGA2ox10*, because the Arabidopsis genes were recognized as *GA2ox* only after our study was initiated (Lange et al., 2020), and because the genes were found in a distinct clade from the known GA2ox proteins. In Arabidopsis, *AtGA2ox9* and *AtGA2ox10* are required for full freezing tolerance and to limit seed number in the developing silique, respectively (Lange et al., 2020). As potential Class III GA2ox enzymes, restricted expression of *MdGA2ox8A* and *-8B* may contribute to limiting diffusion of GA12 and/or other C20 GAs.

This study provides a large amount of preliminary data with both fundamental and practical utility. The apparently simple genomic organization, comprising eight pairs of duplicated genes at homeologous positions, provides an accessible model to study gene expression diversification in a perennial woody plant species with recent whole-genome duplication. Apple seedlings can be grown quickly in controlled environment chambers and greenhouses, and apple seeds are easily obtained from fruit in markets. Many traits influenced by GAs have important production implications in tree fruit crops, including shoot elongation, plant stature, flowering, juvenility, and fruit size, and this work should greatly facilitate further studies to characterize mechanisms of GA signaling in these crops. Finally, the genes identified here are attractive candidates for genetic loci identified as influencing these traits, and the work should enable development of cultivars better suited to emerging production conditions, including those related to high-density management, mechanization, and resilience to climate change.

A limitation of this study is that expression of the genes was analyzed in bulk in entire structures (e.g., petiole, leaf, etc.), which are expected to comprise many distinct cell types, each of which may express specific *GA2ox* genes in a distinct pattern. Understanding the tissue-level expression patterns, and how these are distinct from or overlap with known routes of GA trafficking, is especially important. Future research should extend this genomic/transcriptional study through high-resolution approaches, including *in situ* RNA hybridization with gene-specific probes, and through single-cell RNA sequencing.

## Data availability statement

The datasets presented in this study can be found in online repositories. The names of the repository/repositories and accession number(s) can be found in the article/**Supplementary Material**.

## Author contributions

SZ and SVN designed the research. SZ, CG and SVN performed the research and analyzed the data; SZ drafted the manuscript. SVN composed the final manuscript copy. All authors contributed to the article and approved the submitted version.

## Supplementary material

Supplementary File 1 Protein sequences from Arabidopsis, rice, pea and bean cataloged as “Gibberellin 2-beta-dioxygenase” in the ExPASy Enzyme Database (fasta formatted file).

Supplementary File 2 Protein sequences in the ‘GDDH13’ genome annotation with high homology to the canonical GA2ox protein sequences cataloged in the ExPASy Enzyme Database as shown in **Supplementary File 1** (fasta formatted file).

Supplementary File 3 Arabidopsis protein sequences with high homology to the GDDH13 sequences shown in **Supplementary File 2** (fasta formatted file).

Supplementary File 4 HMMer protein structure analysis results.

Supplementary File 5 Sequences of transcript isoforms at each *MdGA2ox* locus derived from the ‘Gala’ shoot apex based on the ‘GDDH13’ genome annotation (fasta formatted file).

Supplementary File 6 Coding sequences of apple *GA2ox* gene models as annotated in the ‘Gala’ reference genome (fasta formatted file).

Supplementary File 7 TaqMan™ gene expression assays for apple *GA2ox* and *ACTIN* genes.

Supplementary File 8 Sequence alignment of the 16 GA2ox protein sequences from apple and other plants as shown in . Several sequences (MdGA2ox3A, -4B, -6A, -7A and -7B) were curated based on our transcriptome data and the ‘Gala’ genome. Conserved active site amino acids of the 2OG-FEII_Oxy domain catalytic core that interact with 2-oxoglutarate and Fe^2+^ are highlighted in the consensus sequence in yellow and red, respectively, and denoted under the sequence with an asterisk.
