# Supplementary material for: Conservation and divergence of expression of GA2-oxidase homeologs in apple (Malus x domestica Borkh.)
Source: Front Plant Sci. 2023 Apr 26;14:1117069. doi: 10.3389/fpls.2023.1117069 (PMC10169729; doi:10.3389/fpls.2023.1117069)
Supplement: Supplementary File 1 — Protein sequences from Arabidopsis, rice, pea and bean cataloged as “Gibberellin 2-beta-dioxygenase” in the ExPASy Enzyme Database (fasta formatted file). [file DataSheet_1.zip › Datasheet 1/Supplementary file 7.docx]

**Table S1. TaqMan^TM^ gene expression assays for apple *GA2OX* and *Actin* genes**

| **Gene** | **Gene ID** | **Primer/Probe** | **Sequence (5’-3’)** |
| --- | --- | --- | --- |
| *MdGA2OX1A* | MD05G1207000 | Forward | AGAATGGTGATGTTGGGTGGGTAG |
|  |  | Probe | AGTACCTCCTCCTCACAA |
|  |  | Reverse | CATTCAAAGCAGAACAAAACTCTTCTG |
| *MdGA2OX1B* | MD10G1194100 | Forward | ATTTCTTTGAGAGATGGGAGTTG |
|  |  | Probe | TTCAGTCCCACCTGAT |
|  |  | Reverse | CATTAGTCAAAACCTGTAAAGAGTCAC |
| *MdGA2OX2A* | MD05G1283800 | Forward | CCTGAAATCCAACAACACATCAG |
|  |  | Probe | CCTGCAGATCTGTCTCA |
|  |  | Reverse | GTCATCACCTGCAAGCAATC |
| *MdGA2OX2B* | MD10G126200 | Forward | CACACAGACCCACAGATCATT |
|  |  | Probe | CTGTCCTGAAATCCAAC |
|  |  | Reverse | TTAGTCATCACCTGCAAGCAATC |
| *MdGA2OX3A* | MD09G1286800 | Forward | CTTACAGGCTTTAACGAATGGGAGGT |
|  |  | Probe | TAGTGAGCGTGAGACAT |
|  |  | Reverse | GCAGAGGAGACATCCATGC |
| *MdGA2OX3B* | MD17G1279100 | Forward | TGATATTTCCGACGACCCTACGAAAT |
|  |  | Probe | TCAGTTGTGCAGTGAATG |
|  |  | Reverse | CAAGAACCTCACATGCCAATTCT |
| *MdGA2OX4A* | MD13G1148400 | Forward* | CAAAACCATCTCAAATGAGCCTAC |
|  |  | Probe | AATTCAGCTCGGCAGTGA |
|  |  | Reverse | CACAATCCCTCACCAATCAGAT |
| *MdGA2OX4B* | MD16G1148400 | Forward | CAGCCGAGAGGTCTCAAGTG |
|  |  | Probe | ACACTCATTGTCAAAGCT |
|  |  | Reverse | TGGCAAAGAAGCTGAGGCTTTG |
| *MdGA2OX5A* | MD13G1008700 | Forward | CAAGACGAAGTGGGAGGACTT |
|  |  | Probe* | CAACTCTTGAAAGACTCC |
|  |  | Reverse* | TGGTCATTGCTCCACGCCTG |
| *MdGA2OX5B* | MD16G1006700 | Forward | CTTGTTATGGGGACGGTTTTAGCT |
|  |  | Probe | CTCTCAGGGAAGTGATG |
|  |  | Reverse | GTAGCCCAGGTTGTTCACTAGAAT |
| *MdGA2OX6A* | MD05G1341000 | Forward | GATGAATGGATCGCAGTTAAACCTAATC |
|  |  | Probe | CAGAGGCTCTAGTCATC |
|  |  | Reverse | TGTTGCTCCATGCCTGAAATAGATC |
| *MdGA2OX6B* | MD10G1314500 | Forward | TTCCACTCAACGACATTTCTGCT |
|  |  | Probe | CATCAGCTTCTGGTCTC |
|  |  | Reverse | GTTCCATTGTTGAGCTGAGTGTATG |
| *MdGA2OX7A* | MD03G1210700 | Forward | ATGGTCAGAAGCATTTCATATATCTGTC |
|  |  | Probe | ACCGAAATTCCAACGAT |
|  |  | Reverse | TTGTTGATCTGAGACTGTTGTGATG |
| *MdGA2OX7B* | MD11G1225300+MD11G1225400 | Forward | TCCAACAATGAATGATCTTCACAAGAGT |
|  |  | Probe | CAAGTCAACAATTGAAGC |
|  |  | Reverse | CAAAGGCTGTGCCAGAATTTCAG |
| *MdACTIN7* | MD01G1001600 | Forward | TGAAGGCTGGATTTGCTGGTG |
|  |  | Probe | ATGATGCTCCCAGGGCT |
|  |  | Reverse | GGTAAGGATACCTCTTTTCGACTGTGC |

Note: * Oligo sequences that were curated based on ‘Gala’ shoot apex transcriptome reads. Additional primers (5’-3’) used to confirm transcript models at the *MdGA2OX7B* locus include: 1) 5’-UTR region, forward primer 1 (GGATTGTAAAAGTTGTGGATGGT) or forward primer 2 (CTTTTCTACGATACAAGCGATAT) and reverse primer (TAGTACTGCCTTGGAATCTG); 2) ORF region of the MD11G1225300 transcript, forward primer (ATGGATTTCGAACCTCCATTCC) and reverse primer (TTAAAGGTGAAATAGGAAAAGTACGT); 3) ORF region of the MD11G1225400 transcript, forward primer (TTGGTTTTTAGTTGTCAAAAAAGC) and reverse primer (GATATGTTTTTCAAAGGAGAAATC); 4) ORF region of the intact long transcript at *MdGA2OX7B*, forward primer for the MD11G1225300 transcript and reverse primer for the MD11G1225400 transcript.
