# Supplementary figures and images for: Conservation and divergence of expression of GA2-oxidase homeologs in apple (Malus x domestica Borkh.)
Source: Front Plant Sci. 2023 Apr 26;14:1117069. doi: 10.3389/fpls.2023.1117069 (PMC10169729; doi:10.3389/fpls.2023.1117069)

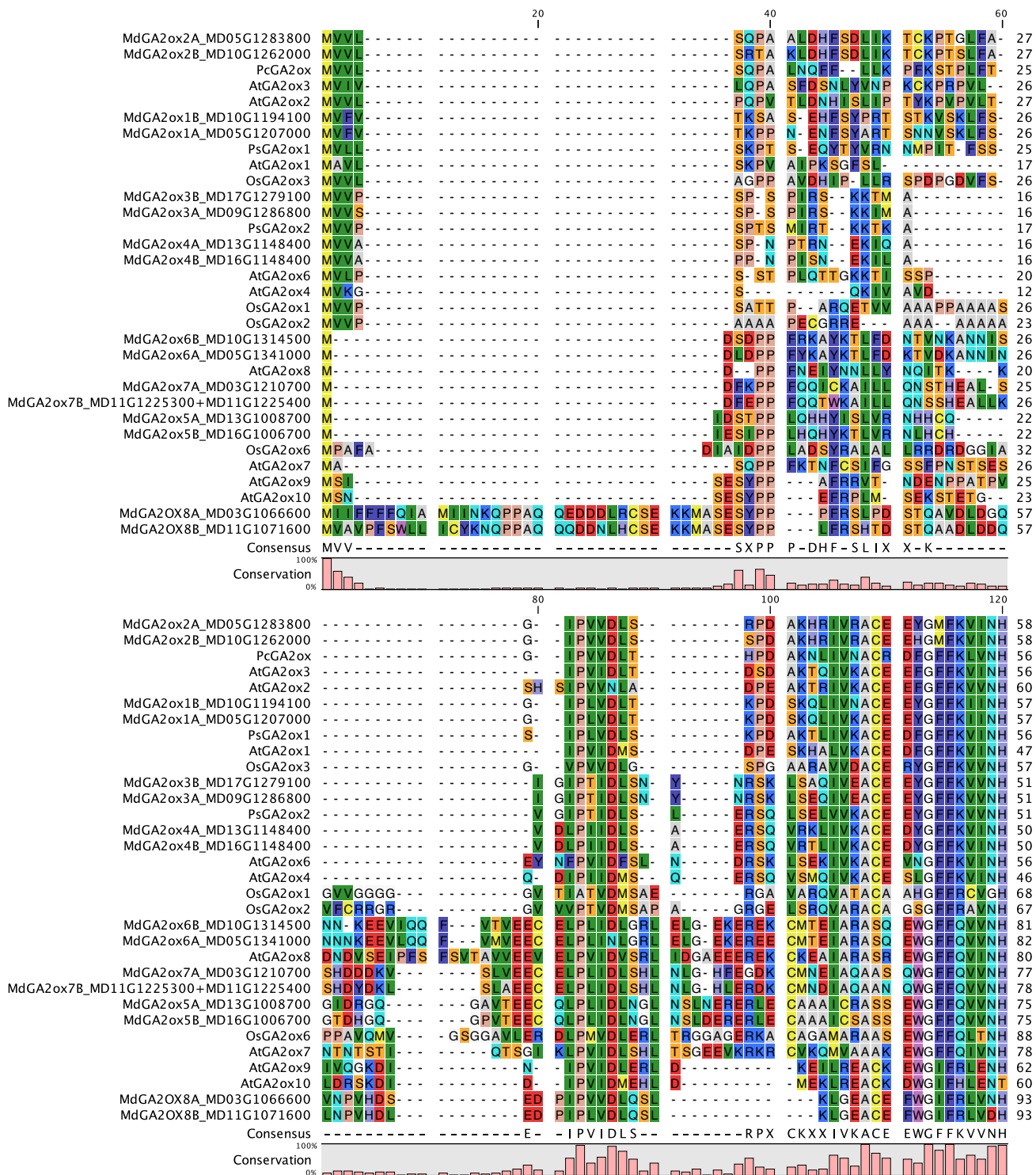

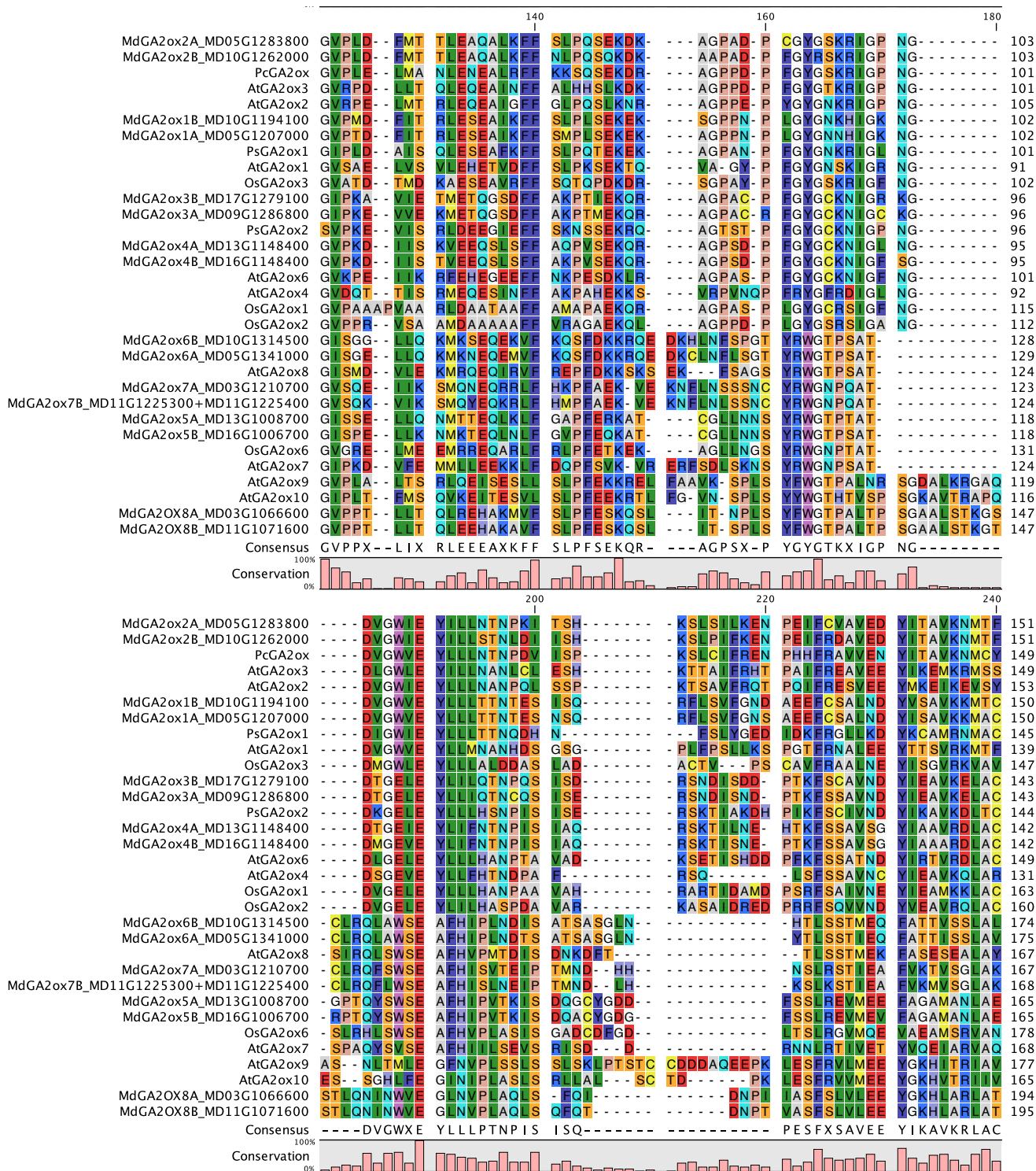



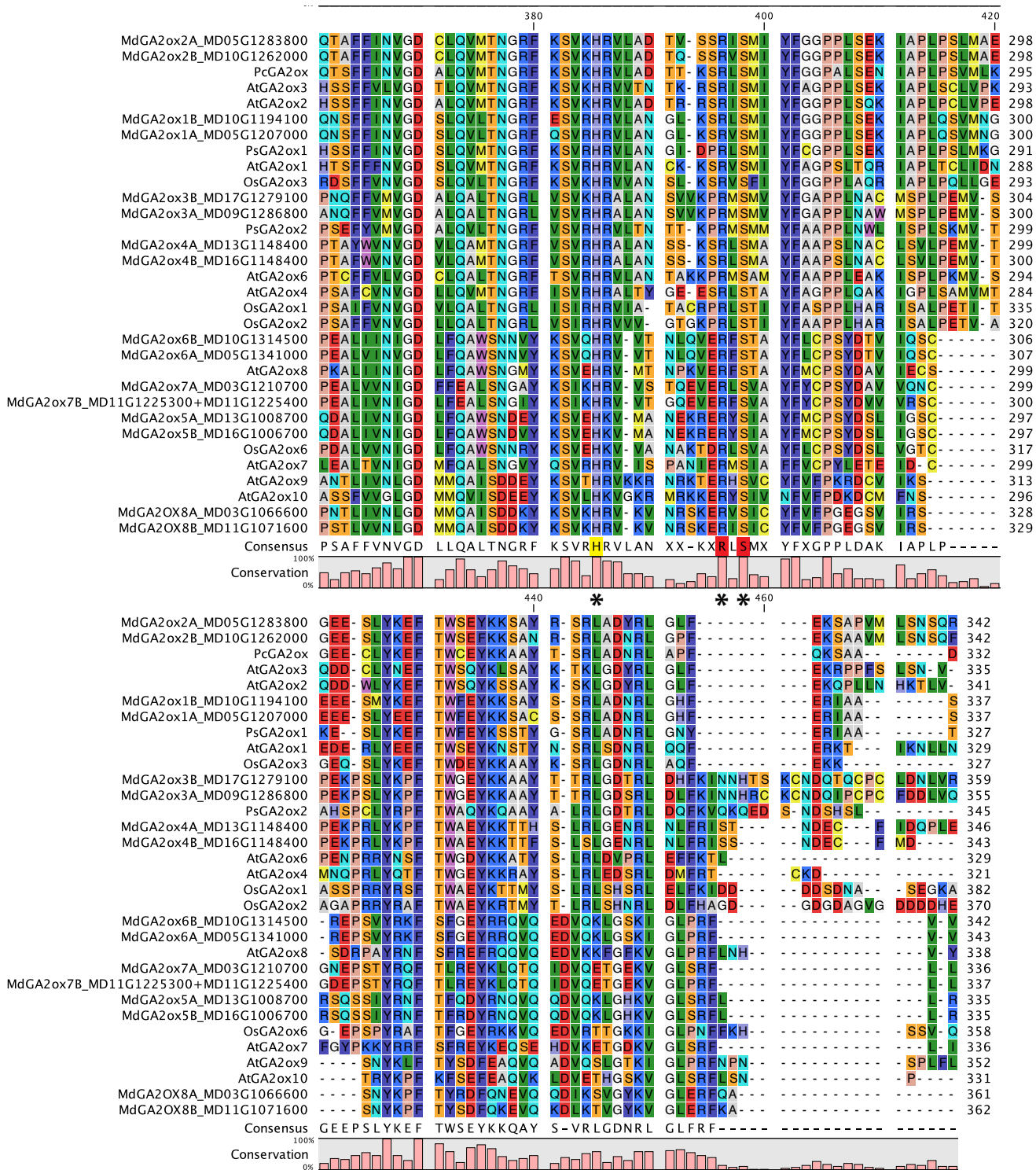

Supplement: Supplementary File 1 — Protein sequences from Arabidopsis, rice, pea and bean cataloged as “Gibberellin 2-beta-dioxygenase” in the ExPASy Enzyme Database (fasta formatted file). [file DataSheet_1.zip › Datasheet 1/Supplementary file 8.pdf]
